# A prognostic nomogram to predict the cancer-specific survival of patients with initially diagnosed metastatic gastric cancer: a validation study in a Chinese cohort

**DOI:** 10.1007/s12094-024-03576-4

**Published:** 2024-06-25

**Authors:** Ziming Zhao, Erxun Dai, Bao Jin, Ping Deng, Zulihaer Salehebieke, Bin Han, Rongfan Wu, Zhaowu Yu, Jun Ren

**Affiliations:** 1 Department of General Surgery, Northern Jiangsu People’s Hospital, Clinical Medical School, Yangzhou University, Yangzhou, People’s Republic of China; 2 Department of General Surgery, Xinyuan County People’s Hospital, Ili Kazak Autonomous Prefecture, People’s Republic of China; 3 Department of Oncology, Northern Jiangsu People’s Hospital, Clinical Medical School, Yangzhou University, Yangzhou, People’s Republic of China; 4 Department of General Surgery, General Surgery Institute of Yangzhou, Northern Jiangsu People’s Hospital, Yangzhou, People’s Republic of China; 5 Department of General Surgery, Northern Jiangsu People’s Hospital Affiliated to Yangzhou University, Yangzhou, People’s Republic of China

**Keywords:** Metastatic gastric cancer, Nomogram, Cancer-specific survival, Nomogram-based prediction, Chinese patients

## Abstract

**Background:**

Few studies have been designed to predict the survival of Chinese patients initially diagnosed with metastatic gastric cancer (mGC). Therefore, the objective of this study was to construct and validate a new nomogram model to predict cancer-specific survival (CSS) in Chinese patients.

**Methods:**

We collected 328 patients with mGC from Northern Jiangsu People’s Hospital as the training cohort and 60 patients from Xinyuan County People’s Hospital as the external validation cohort. Multivariate Cox regression was used to identify risk factors, and a nomogram was created to predict CSS. The predictive performance of the nomogram was evaluated using the consistency index (C-index), the calibration curve, and the decision curve analysis (DCA) in the training cohort and the validation cohort.

**Results:**

Multivariate Cox regression identified differentiation grade (*P* < 0.001), T-stage (*P* < 0.05), N-stage (*P* < 0.001), surgery (*P* < 0.05), and chemotherapy (*P* < 0.001) as independent predictors of CSS. Nomogram of chemotherapy regimens and cycles was also designed by us for the prediction of mGC. Thus, these factors are integrated into the nomogram model: the C-index value was 0.72 (95% CI 0.70–0.85) for the nomogram model and 0.82 (95% CI 0.79–0.89) and 0.73 (95% CI 0.70–0.86) for the internal and external validation cohorts, respectively. Calibration curves and DCA also demonstrated adequate fit and ideal net benefit in prediction and clinical applications.

**Conclusions:**

We established a practical nomogram to predict CSS in Chinese patients initially diagnosed with mGC. Nomograms can be used to individualize survival predictions and guide clinicians in making therapeutic decisions.

## Background

Although the incidence of gastric cancer (GC) is decreasing, it remains the fourth leading cause of cancer-related deaths. Globally, more than 1,000,000 new cases are diagnosed each year, and the number of deaths from GC is about 768,800 each year; in China, there are approximately 477,800 new cases and 373,200 deaths each year [1–3]. The 5-year survival rate for patients with GC with distant metastasis is less than 5% and the median survival time is 11–18 months [4, 5]. Treatment of GC depends mainly on the stage of the tumor, and for non-metastatic gastric cancer, attention should be paid to supplementing adjuvant chemotherapy or radiation therapy while applying surgical treatment, and a combination of therapeutic methods is often adopted for mGC [6–8].

Despite the great progress in lymph node dissection, neoadjuvant chemoradiotherapy, targeted therapy, and immunotherapy, the rate of early GC diagnosis is very low due to the lack of typical clinical symptoms in early GC, and most patients are already in an advanced stage at the time of diagnosis, especially in China, where almost 80% of the patients with GC are diagnosed with advanced or metastatic disease [9–11]. The assessment of mortality risk in patients with GC is still based on the TNM classification system of the American Joint Committee on Cancer (AJCC) [12]. In particular, there is significant heterogeneity among GC patients in terms of demographic and clinicopathological characteristics, such as age, gender, body mass index, tumor size, differentiation grade, metastatic organ sites and number, as well as applied treatment regimens, and clinicopathological characteristics, which may affect the prognosis of the patients; however, they are not reflected in the TNM classification system. Good clinical decision-making, clinical trial design, and an accurate prognosis are needed to predict high-risk patient groups. The use of nomograms is of great clinical help, and there are many nomograms that have been produced for the diagnosis of the disease, and perform better than the TNM classification system of the AJCC [13–15]. Although there have been many nomograms based on different databases to predict the survival of individuals with GC, few studies have been designed to predict the survival of Chinese patients initially diagnosed with mGC [16–20]. Therefore, the objective of this study was to construct and validate a new nomogram model to predict CSS in Chinese patients with mGC.

## Materials and methods

### Patients and data collection

We collected 328 patients with mGC diagnosed for the first time at Northern Jiangsu People’s Hospital from January 2012 to December 2022 as a training cohort. Based on the same criteria, 60 patients at Xinyuan County People’s Hospital from January 2018 to December 2022 were collected as an external validation cohort.

**Fig. 1:**
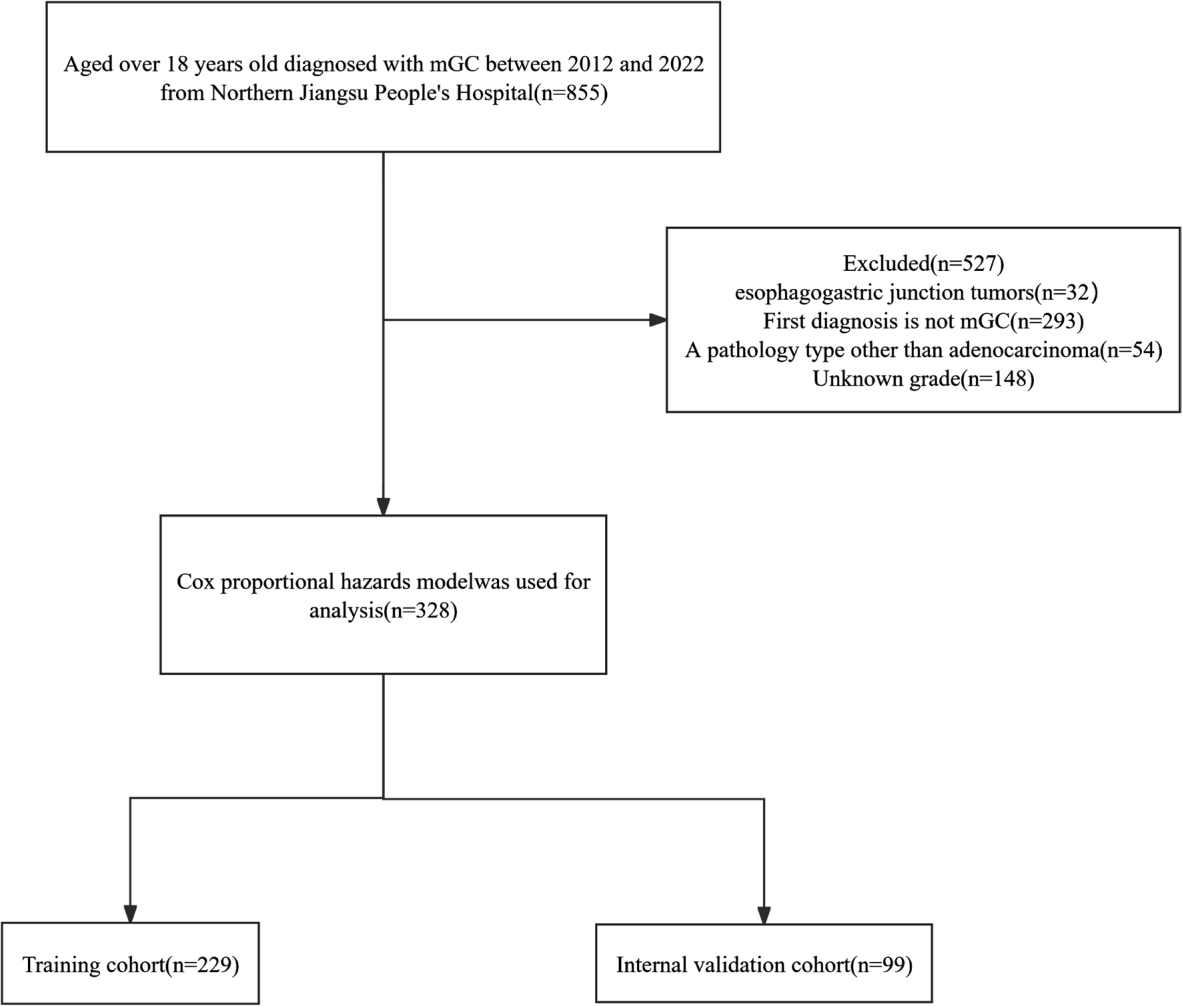
Flow chart of patient inclusion. mGC, metastatic gastric cancer

Inclusion criteria for this study: (1) histological diagnosis of gastric adenocarcinoma; (2) age ≥ 18 years; (3) inclusion of patients initially diagnosed with distant organ metastasis and/or lymph node metastasis based on the AJCC staging system; (4) availability of complete hospitalization and follow-up data; and (5) the ultimate cause of death was sex-related death related to gastric cancer. Exclusion criteria: (1) age < 18 years; (2) comorbidity with other tumors; and (3) lack of or incomplete clinical data, including information on the depth of tumor infiltration (T-stage) lymph node metastasis (N-stage) and distant metastasis (M-stage), and distant organ metastases of gastric cancer (Fig. 1). The TNM staging was re-staged according to the AJCC 8th edition staging system.

Finally, the collected 328 patients were randomly divided into two cohorts according to 7:3, one group was a modeling cohort of 229 patients for studying CSS and the construction of nomogram models, and the other group was an internal validation cohort of 99 patients to validate the nomogram models. The 60 patients collected from Xinyuan County People’s Hospital were used as the external validation sequence. All gastric cancers were initially diagnosed by computed tomography or positron emission tomography computed tomography with at least one distant metastasis, including the peritoneum, liver, lung, bone, brain, kidney, pancreas, spleen and ovary. The ethics committees of the Northern Jiangsu People’s Hospital and the Xinyuan County People’s Hospital had approved the study.

### Follow-up and outcome

Follow-up methods included medical records and telephone follow-up. Observations were made every 1 month for the first 6 months after treatment, every 3 months for the next 6 months, every 6 months for 2–3 years, and annually thereafter. Each follow-up visit included physical examination, laboratory tests, and chest/abdominal/pelvic enhancement CT. The last follow-up date was December 2022.

### Endpoint definition

GC-specific death was defined as death from GC as the underlying cause according to our data, and the endpoint of the current study was CSS, which was the interval between the initial diagnosis of GC and the occurrence of GC-specific death.

### Statistical analysis and nomogram construction

**Fig. 2:**
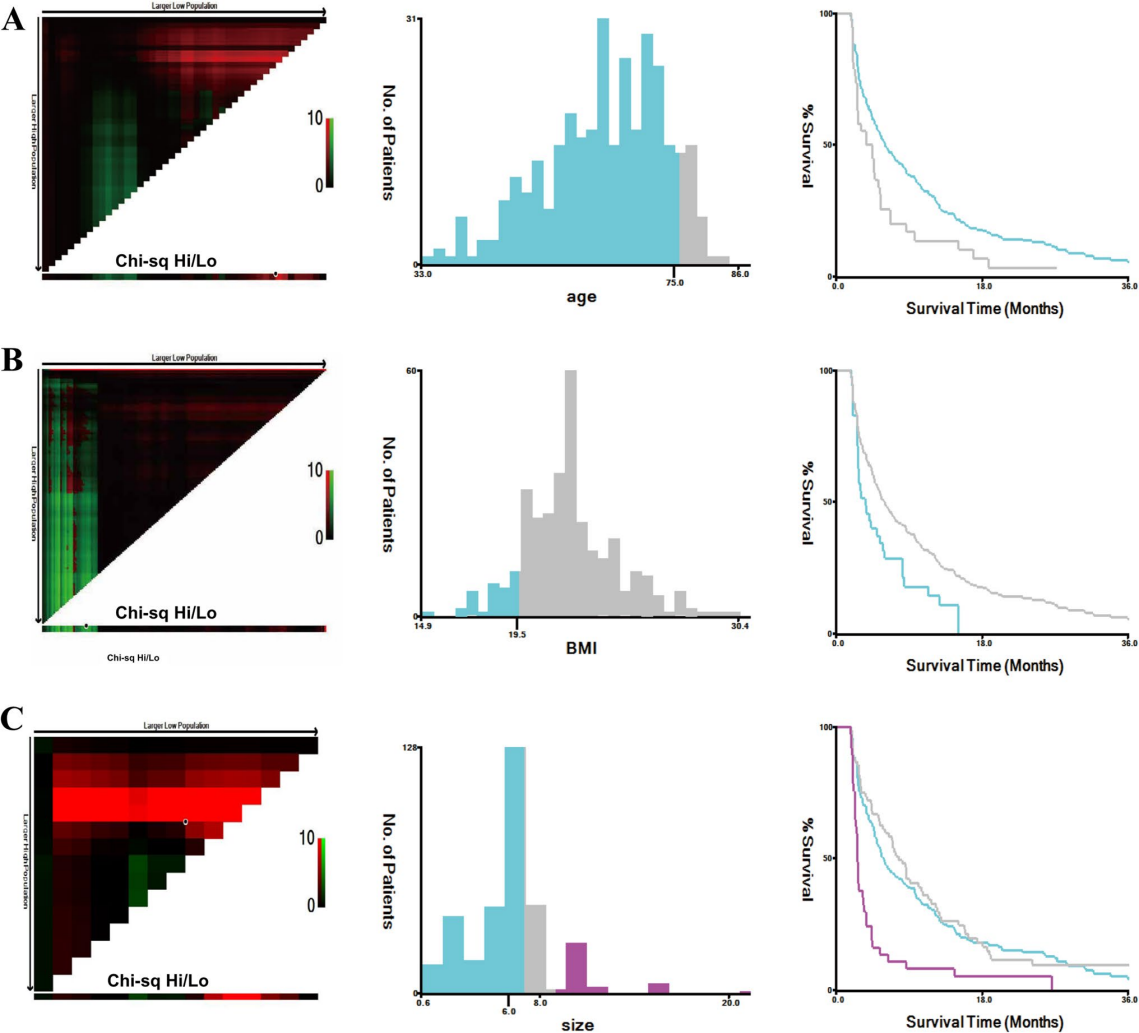
**A** The best cutoff value for age at diagnosis was 75 years (*P* < 0.001). **B** The best cutoff value for body mass index was 19.5 kg/m^2^ (*P* < 0.001). **C** The optimal cutoff values for tumor size were 6.0 cm and 8.0 cm (*P* < 0.001)

**Fig. 3:**
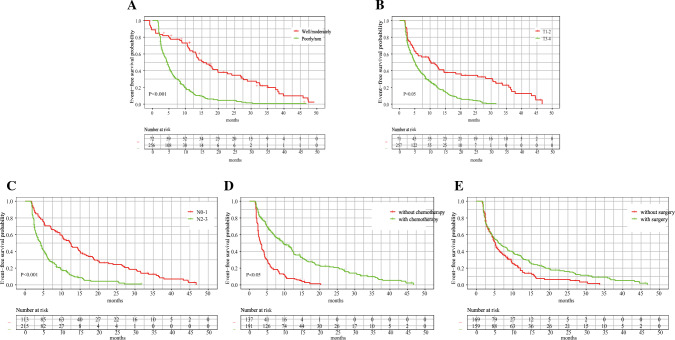
Survival curves for each clinical variable after multifactorial analysis. **A** Survival curves for degree of tumor differentiation. **B** Survival curves for T-stage (according to the AJCC 8th edition staging system). **C** Survival curves for N-stage (according to the AJCC 8th edition staging system). **D** Survival curves for surgery. **E** Survival curves for chemotherapy

We use X-tile software to determine the optimal cutoff value for the stratification of continuous variables, such as age, body mass index (BMI), and tumor size, the risk value that would stratify cancer-related deaths. Taking into account the significant effect of age on survival risk, age was divided into two groups based on the optimal cutoff values calculated by the X-tile software with a cutoff of 75 years. Body mass index (BMI) was divided into two groups with a cutoff of 19.5 kg/m^2^. Tumor size was divided into 3 groups with 6.0 cm and 8.0 cm as cutoffs, respectively (Fig. 2). Data were analyzed using SPSS statistical software (version 26.0, IBM, USA), and clinical characteristics were described as percentages, medians, and interquartiles (IQR). Comparisons of categorical variables were made using the Chi-square test or Fisher’s exact test. A two-tailed *P* < 0.05 was considered statistically significant. Significant variables from the univariate analysis were included in the multivariate Cox regression to identify independent factors. Survival curves of the independent factors were plotted using Kaplan–Meier analysis (Fig. 3). Based on the final multivariate Cox regression, the nomogram was constructed using the rms and survival packages in R language (version 4.3.2, http://www.r-project.org).

The training cohort was randomly divided into two groups by 7:3 using R. The modeling group was used to build the nomogram model, the internal and external validation groups were used to validate the model. Consistency index (C-index), calibration curve and decision curve analysis (DCA) were used to assess the predictive efficacy of the nomogram. C-index was used to assess the predictive accuracy and discriminative power of the nomogram model. The calibration curve is used to assess the calibration of the model. DCA was used to evaluate clinical utility.

## Results

### Baseline characteristics

According to our inclusion and exclusion criteria (Fig. 1), a total of 328 patients with initial diagnosis of mGC from 2012 to 2022 from the Northern Jiangsu People’s Hospital were finally included in this study. All cases were diagnosed with mGC at the time of initial diagnosis. The mean age of the patients was 63.37 ± 10.175 years. 68.90% of these patients (*N* = 226) were male. The most common site of metastasis was the liver (51.52%) followed by the peritoneum (46.65%), lung (7.62%) and bone (7.01%). Table 1 shows the characteristics of the patients. All eligible cases were randomly assigned to the training cohort (229, 70%) or the internal validation cohort (99, 30%) to create and validate the prognostic nomogram; the cohort from Xinyuan County People’s Hospital was used for external validation. The median CSS was 9.4 months [95% CI 8.03–10.77] and 11.6 months [95% CI 8.84–14.33] in the training cohort and internal validation cohort, respectively, and the median CSS was 11.7 months [95% CI 9.18–14.32] for the external cohort, and Table 2 summarizes the characteristics of the patients in the two cohorts. The treatment strategy for all patients was developed by a multidisciplinary team of surgeons, medical oncologists, and radiologists from the Northern Jiangsu People’s Hospital. Clinical staging was further confirmed by experienced radiologists, and chemotherapy regimens used for patients with metastatic gastric cancer mainly included mFLOT and FOLFOX (or its derivatives, such as SOX or XELOX). (1) FOLFOX: Oxaliplatin 85 mg/m^2^ + Fluorouracil 2800 mg/m^2^ continuous intravenous injection over 48 h; every 2 weeks; (2) SOX: Oxaliplatin 130 mg/m^2^ intravenous injection + Tegafur Gimeracil Oteracil Potassium Capsule 40–60 mg bid D1–D14; every 3 weeks; (3) XELOX: Oxaliplatin 130 mg/m^2^ + Capecitabine 1000 mg/m^2^ bid D1–D14; every 3 weeks; and (4) mFLOT: Docetaxel 50–60 mg/m^2^ + Oxaliplatin 85 mg/m^2^ + Fluorouracil 2800 mg/m^2^ intravenous injection over 48 h; every 2 weeks (Table 3).

**Table 1:** The death in patients with mGC in Northern Jiangsu People’s Hospital

Characters	Total patients		Death		No death	
	*n* = 328		*n* = 284		*n* = 44	
	No. of patients	%	No. of patients	%	No. of patients	%
Age (years)						
< 75	281	85.67	242	85.21	39	88.64
≥ 75	47	14.33	42	14.79	5	11.36
Gender						
Male	226	68.90	199	70.07	27	61.36
Female	102	31.10	85	29.93	17	38.64
BMI, kg/m^2^						
< 19.5 kg/m^2^	35	10.67	31	10.92	4	9.09
≥ 19.5 kg/m^2^	293	89.33	253	89.08	40	90.91
Tumor location						
Cardia	104	31.71	97	34.16	7	15.91
Middle	122	37.20	95	33.45	27	61.36
Distal	93	28.35	85	29.93	8	18.18
Overlapping/NOS	9	2.74	7	2.46	2	4.55
Tumor size (cm)						
< 6 cm	117	35.67	102	35.91	15	34.09
6–8 cm	128	39.02	108	38.03	20	45.46
≥ 8 cm	83	25.30	74	26.06	9	20.45
Grade						
Well/moderately differentiated	72	21.95	58	20.42	14	31.82
Poorly/non differentiated	256	78.05	226	79.58	30	68.18
T stage						
T1, T2	71	21.65	59	20.77	12	27.27
T3, T4	257	78.35	225	79.23	32	72.73
N stage						
N0, N1	113	3445.00	98	34.51	15	34.09
N2, N3	215	65.55	186	37.32	29	65.91
Brain metastases						
No	327	99.70	283	99.65	44	100.00
Yes	1	0.30	1	0.35	0	0.00
Bone metastases						
No	305	92.99	263	92.61	42	95.45
Yes	23	7.01	21	7.39	2	4.55
Lung metastases						
No	303	92.38	263	92.61	40	90.91
Yes	25	7.62	21	7.39	4	9.09
Liver metastases						
No	159	48.48	146	51.41	13	29.55
Yes	169	51.52	138	48.59	31	70.45
Pancreatic metastases						
No	323	98.48	283	99.65	44	100.00
Yes	5	1.52	1	0.35	0	0.00
Splenic metastases						
No	322	98.17	278	97.89	44	100.00
Yes	6	1.83	6	2.11	0	0.00
Ovarian metastases						
No	321	97.87	277	97.54	44	100.00
Yes	7	2.13	7	2.46	0	0.00
Peritoneal metastases						
No	175	53.35	147	51.76	28	63.64
Yes	153	46.65	137	48.24	16	36.36
Number of transfers						
1	270	82.32	235	82.75	35	79.55
2	55	16.77	46	16.20	9	20.45
3	3	0.91	3	1.05	0	0.00
Surgery						
No	169	51.52	137	48.24	32	72.73
Yes	159	48.48	147	51.76	12	27.27
Chemotherapy						
No	137	41.77	132	46.48	5	11.36
Yes	191	58.23	152	53.52	39	88.64
Radiotherapy						
No	324	98.78	280	98.59	44	100.00
Yes	4	1.22	4	1.41	0	0.00
Targeted therapy						
No	311	94.82	268	94.37	44	100.00
Yes	17	5.18	16	5.63	0	0.00
Immunotherapy						
No	251	76.52	237	83.45	14	31.82
Yes	77	23.48	47	16.55	30	68.18
CHIP						
No	314	95.73	272	95.77	42	95.45
Yes	14	4.27	12	4.23	2	4.55

*NOS* not otherwise specified, *CHIP* chemotherapeutic hyperthermic intraperitoneal perfusion

**Table 2:** Basic information of the training, internal validation, and external validation set

Characters	Training cohort		Internal validation cohort		External validation cohort	
	*n* = 229		*n* = 99		*n* = 60	
	No. of patients	%	No. of patients	%	No. of patients	%
Age (years)						
< 75	191	83.41	90	90.91	54	90.00
≥ 75	38	16.59	9	9.09	6	10.00
Gender						
Male	152	66.38	74	74.75	47	78.33
Female	77	33.62	25	25.25	13	21.67
BMI, kg/m^2^						
< 19.5 kg/m^2^	26	11.35	9	9.09	17	28.33
≥ 19.5 kg/m^2^	203	88.65	90	90.91	43	71.67
Tumor location						
Cardia	81	35.37	23	23.23	29	48.33
Middle	77	33.62	45	45.45	14	23.33
Distal	63	27.51	30	30.30	12	20.00
Overlapping/NOS	8	3.49	1	1.01	5	8.34
Tumor size (cm)						
< 6 cm	86	37.55	31	31.31	33	55.00
6–8 cm	85	37.12	43	43.43	4	6.67
≥ 8 cm	58	25.33	25	25.25	23	38.33
Grade						
Well/moderately differentiated	50	21.83	22	22.22	15	25.00
Poorly/non differentiated	179	78.17	77	77.78	45	75.00
T stage						
T1, T2	49	21.40	22	22.22	10	16.67
T3, T4	180	78.60	77	77.78	50	83.33
N stage						
N0, N1	84	36.68	29	29.29	12	20.00
N2, N3	145	63.32	70	70.71	48	80.00
Brain metastases						
No	228	99.56	99	100.00	60	100.00
Yes	1	0.44	0	0.00	0	0.00
Bone metastases						
No	214	93.45	91	91.92	49	81.67
Yes	15	6.55	8	8.08	11	18.33
Lung metastases						
No	210	91.70	93	93.94	44	73.33
Yes	19	8.30	6	6.06	16	26.67
Liver metastases						
No	110	48.03	49	49.49	19	31.67
Yes	119	51.97	50	50.51	41	68.33
Pancreatic metastases						
No	226	98.69	97	97.98	58	96.67
Yes	3	1.31	2	2.02	2	3.33
Splenic metastases						
No	225	98.25	97	97.98	58	96.67
Yes	4	1.75	2	2.02	2	3.33
Ovarian metastases						
No	223	97.38	98	98.99	59	98.33
Yes	6	2.62	1	1.01	1	1.67
Peritoneal metastases						
No	124	54.15	51	51.52	43	78.33
Yes	105	45.85	48	48.48	17	21.67
Number of transfers						
1	189	82.53	81	81.82	32	53.33
2	37	16.16	18	18.18	23	38.33
3	3	1.31	0	0.00	5	8.34
Surgery						
No	123	53.71	46	46.46	38	63.33
Yes	106	46.29	53	53.54	22	36.67
Chemotherapy						
No	94	41.05	43	43.43	34	56.67
Yes	135	58.95	56	56.57	26	43.33
Radiotherapy						
No	225	98.25	99	100.00	52	86.67
Yes	4	1.75	0	0.00	8	13.33
Targeted therapy						
No	219	95.63	92	92.93	43	71.67
Yes	10	4.37	7	7.07	17	28.33
Immunotherapy						
No	179	78.17	72	72.73	40	66.67
Yes	50	21.83	27	27.27	20	33.33
CHIP						
No	217	94.76	97	97.98	47	78.33
Yes	12	5.24	2	2.02	13	21.67

*NOS* not otherwise specified, *CHIP* chemotherapeutic hyperthermic intraperitoneal perfusion

**Table 3:** Chemotherapy information of the training set, internal validation set, and external validation set

Chemotherapy regimens	Training cohort		Internal validation cohort		External validation cohort	
	*n* = 135		*n* = 56		*n* = 26	
	No. of patients	%	No. of patients	%	No. of patients	%
Platin-based doublet regimen						
FOLFOX	6	4.44	3	5.36	0	0.00
SOX	32	23.70	14	25.00	11	42.31
XELOX	20	14.81	6	10.71	6	23.08
Taxanes contained regimen						
mFLOT	18	13.33	8	14.29	5	19.23
Docetaxel plus fluorouracil	12	8.89	5	8.93	1	3.85
Docetaxel plus S-1 capsule	7	5.19	6	10.71	0	0.00
Monotherapy						
S-1 capsule	29	21.48	9	16.07	2	7.69
Capecitabine	11	8.15	5	8.93	1	3.85

(1) FOLFOX: Oxaliplatin 85 mg/m^2^ + Fluorouracil 2800 mg/m^2^ continuous intravenous injection over 48 h; every 2 weeks; (2) SOX: Oxaliplatin 130 mg/m^2^ intravenous injection + Tegafur Gimeracil Oteracil Potassium Capsule 40–60 mg bid D1–D14; every 3 weeks; (3) XELOX: Oxaliplatin 130 mg/m^2^ + Capecitabine 1000 mg/m^2^ bid D1–D14; every 3 weeks; and (4) mFLOT: Docetaxel 50–60 mg/m^2^ + Oxaliplatin 85 mg/m^2^ + Fluorouracil 2800 mg/m^2^ intravenous injection over 48 h; every 2 weeks

### Independent prognostic factors in the training cohort

Univariate analysis showed that differentiation grade, clinical T stage, N stage, liver metastasis, peritoneal metastasis, surgery, chemotherapy, and immunotherapy were associated with the prognosis of the patient. Factors with a *P* value < 0.05 in the univariate analysis were included in the multivariate Cox regression model. Multivariate analysis identified differentiation grade (*P* < 0.001), T-stage (*P* < 0.05), N-stage (*P* < 0.001), surgery (*P* < 0.05), and chemotherapy (*P* < 0.001) as independent predictors of CSS, and these were included in the prediction model (Table 4).

**Table 4:** Cox univariate and multivariate proportional hazards regression analyses of each factor’s using the training cohort

Characters	No. of patients	CSS (%)	Univariate analysis			Multivariate analysis		
			Hazard ratio	95% CI	*P*	Hazard ratio	95% CI	*P*
Age (years)			1.364	0.945–1.970	0.097			
< 75	191	89.01						
≥ 75	38	92.11						
Gender			0.828	0.615–1.114	0.212			
Male	152	92.11						
Female	77	84.42						
BMI, kg/m^2^			0.699	0.451–1.083	0.109			
< 19.5 kg/m^2^	26	88.46						
≥ 19.5 kg/m^2^	203	89.66						
Tumor location			0.929	0.790–1.092	0.371			
Cardia	81	93.83						
Middle	77	83.12						
Distal	63	92.06						
Overlapping/NOS	8	87.50						
Tumor size (cm)			1.150	0.954–1.387	0.142			
< 6 cm	86	88.37						
6–8 cm	85	88.24						
≥ 8 cm	58	93.10						
Grade			3.909	2.675–5.711	< 0.001			
Well/moderately differentiated	50	82.00				1		
Poorly/non differentiated	179	91.62				2.401	1.541–3.743	< 0.001*
T stage			2.332	1.586–3.429	< 0.001			
T1,T2	49	83.67				1		
T3,T4	180	91.11				1.826	1.225–2.721	0.003*
N stage			2.957	2.141–4.084	< 0.001			
N0,N1	84	88.10				1		
N2,N3	145	90.34				1.803	1.271–2.558	< 0.001*
Brain metastases			0.899	0.126–6.426	0.915			
No	228	89.47						
Yes	1	100.00						
Bone metastases			1.215	0.715–2.062	0.472			
No	214	88.79						
Yes	15	100.00						
Lung metastases			0.863	0.531–1.402	0.552			
No	210	89.05						
Yes	19	94.74						
Liver metastases			0.707	0.537–0.930	0.013			
No	110	94.55				1		
Yes	119	84.87				0.960	0.666–1.384	0.827
Pancreatic metastases			1.266	0.404–3.966	0.686			
No	226	89.38						
Yes	3	100.00						
Splenic metastases			1.537	0.570–4.144	0.396			
No	225	89.33						
Yes	4	100.00						
Ovarian metastases			1.475	0.640–3.397	0.362			
No	223	89.24						
Yes	6	100.00						
Peritoneal metastases			1.438	1.091–1.895	0.009			
No	124	86.29				1		
Yes	105	93.33				1.354	0.936–1.959	0.107
Number of transfers			1.120	0.814–1.540	0.489			
1	189	88.36						
2	37	97.37						
3	3	100.00						
Surgery			0.672	0.504–0.896	0.007			
Yes	106	91.51				1		
No	123	87.80				0.613	0.450–0.835	0.002*
Chemotherapy			0.334	0.248–0.451	< 0.001			
Yes	135	83.70				1		
No	94	97.87				0.384	0.271–0.542	< 0.001*
Radiotherapy			0.672	0.249–1.816	0.434			
Yes	4	100.00						
No	225	89.33						
Targeted therapy			0.814	0.416–1.593	0.548			
Yes	10	90.00						
No	219	89.50						
Immunotherapy			0.515	0.353–0.752	< 0.001			
Yes	50	64.00				1		
No	179	96.65				0.356	0.536–1.251	0.819
CHIP			0.868	0.459–1.641	0.662			
Yes	12	83.33						
No	217	89.86						

*NOS* not otherwise specified, *CHIP* chemotherapeutic hyperthermic intraperitoneal perfusion

### Prognostic nomogram for CSS

**Fig. 4:**
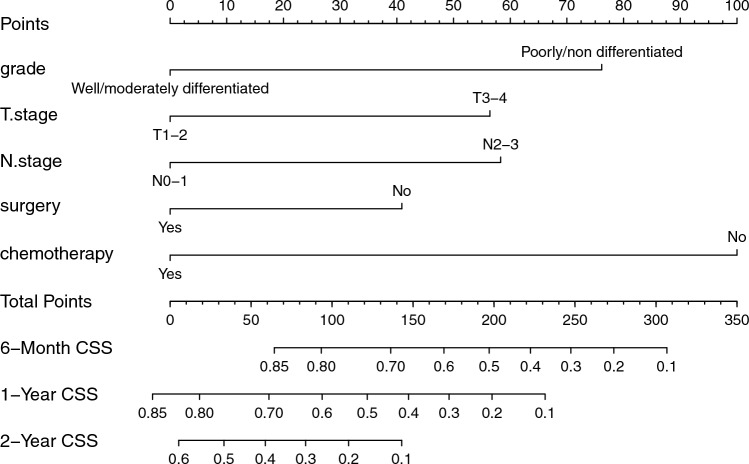
Nomogram predicting the 6-month, 1-year, and 2-year CSS of patients initially diagnosed with mGC

**Fig. 5:**
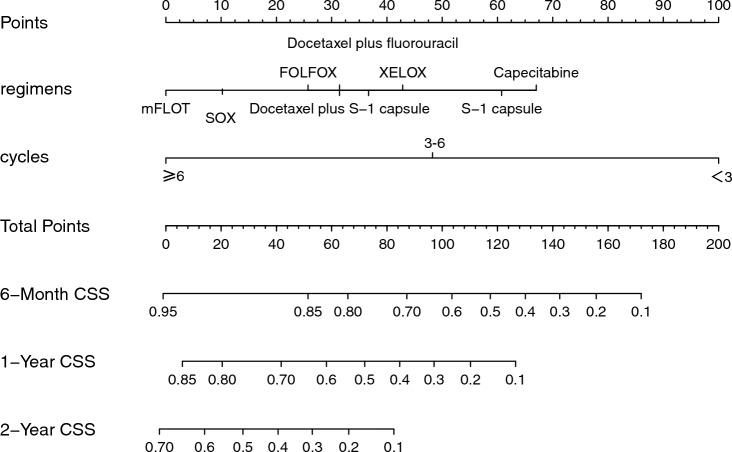
Predicting the chemotherapy regimens and cycles nomogram for 6-month, 1-year, and 2-year CSS for patients with an initially diagnosis of mGC

The predictive model was in the form of a nomogram. Significant independent factors such as differentiation classification, T stage, N stage, surgery, and chemotherapy were used to create the nomogram (Fig. 4). Chemotherapy contributed the most to the prognosis, followed by differentiation grading. The T/N stage, and surgery had a moderate effect on survival. So we created another nomogram about chemotherapy regimens and cycles, which was used to predict the predictive ability of mGC under different chemotherapy regimens and cycles (Fig. 5). Each variable was assigned a score from 0 to 10, which is shown at the top of the column chart; after calculating the total score and positioning it on a scale of total score, a straight line was plotted at different times relative to the date of diagnosis to determine the estimated survival rate.

### Calibration and validation of the nomogram

**Fig. 6:**
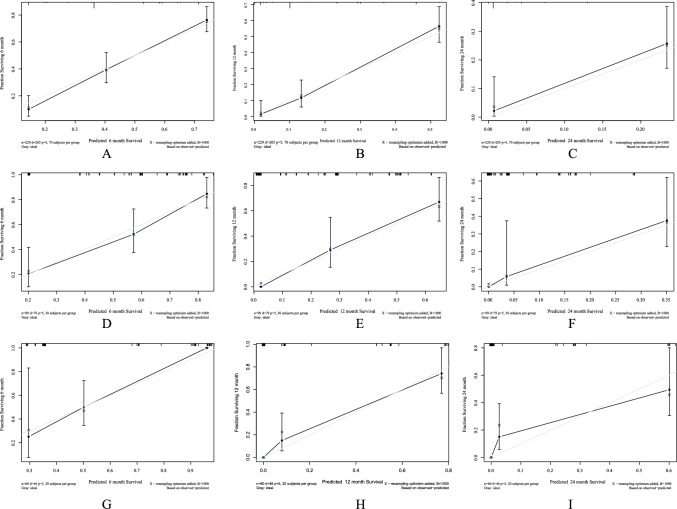
Calibration of the nomogram at 6 months, 1 year, and 2 years in the training, internal validation, and external validation cohorts. The nomogram-predicted CSS is plotted on the *x*-axis and the actual CSS is plotted on the *y*-axis. The 45° line indicates a perfect prediction model. The 6-month, 1-year, and 2-year CSS rates of the (**A**–**C**) training, (**D**–**F**) internal validation, and (**G**–**I**) external validation cohorts

**Fig. 7:**
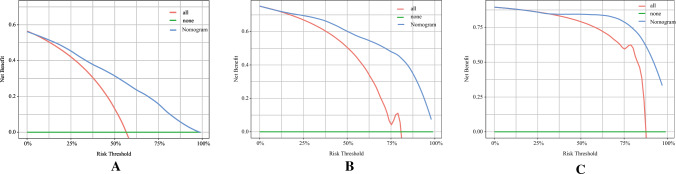
Decision curve analysis of column line graphs for predicting CSS. Based on data from the primary cohort, decision curve analysis was used to determine the clinical utility of column line plots for predicting CSS at the 6-month (**A**), 1-year (**B**), and 2-year (**C**) time points. The horizontal green line corresponds to no cancer-specific deaths, and the solid red line assumes that all patients die of cancer. The blue line represents the net benefit of using the column chart

For the training cohort, the consistency index value was 0.72 (95% CI 0.70–0.85), reflecting the good discriminatory power of the model. The calibration curves also show good agreement in terms of observations and nomogram predictions regarding CSS at 6 months, 1 year, and 2 years (Fig. 6A–C). For the internal validation cohort, with a c-index value of 0.82 (95% CI 0.79–0.89), and the calibration plots showed acceptable agreement between the predictions of the nomogram and observations with respect to CSS at 6 months, 1-year, and 2-year CSS (Fig. 6D–F). For the external validation cohort, the value of the c-index was 0.73 (95% CI 0.70–0.86) (Fig. 6G–I). CSS based on DCA, the nomogram model showed clinical utility at different time points. Compared to the traditional AJCC staging system, it has been confirmed that clinical net benefits for nomogram model at the time endpoint of 6-month, 1-year, and 2-year CSS were better, with a wide range of threshold probabilities in both training (Fig. 7A–C). In Fig. 7A–C, the green line represents the net benefit line for “Treat None”, which assumes that no patients are treated and the net benefit is zero. The red curve represents the net benefit of “Treat All”, which assumes that all patients are treated, and shows the net benefit in this extreme case. The blue curve represents the net benefit of the nomogram. As can be seen in Fig. 7A–C, the overall net benefit of the nomogram is superior than all of the patients’ dead scheme or none of the patients’ dead scheme over the entire range (0–1).

## Discussion

Although some nomograms have been established to predict the prognosis of gastric cancer patients, there are few prediction models for mGC patients of Chinese. We established and validated a nomogram to predict CSS in patients with mGC based on their clinicopathological characteristics and treatment regimen. The guidelines of the National Comprehensive Cancer Network recommend chemotherapy as the first-line treatment for patients with mGC, advanced gastric cancer, or recurrent gastric cancer. Chemotherapy can prolong OS (overall survival) and improve quality of life [21]. A meta-analysis suggests that chemotherapy prolongs the OS of patients with mGC by approximately 6.7 months compared with best supportive care (BSC) [22]. A phase III Randomized Clinical Trial suggests that combination therapy was associated with numerically improved overall survival (OS), although statistically insignificant, and a significant progression-free survival (PFS) benefit compared with monotherapy [23]. Our nomogram also showed better CSS with multidrug combination therapy than with monotherapy. Another meta-analysis suggests that S-1-based combination therapy is superior to S-1 monotherapy in terms of overall survival (OS), progression-free survival (PFS), and overall response rate (ORR) [24]. Chemotherapy cycles show in our Nomogram that a long number of cycles is superior to a short number of cycles for metastatic gastric cancer in terms of CSS. A pooled analysis of the OGSG0604 and OGSG1002 trials suggests that the long cycles (≥ 6) of Docetaxel plus S − 1 capsule (DS) therapy may prolong prognosis [25]. This is also consistent with the Nomogram results we produced.

Currently, the preoperative clinical staging of gastric cancer patients is based on the classification of TNM, which is the most widely used gastric cancer staging system for staging gastric cancer patients, evaluating prognosis, and formulating treatment plans [26]. In the primary lesion of gastric cancer, the aggressiveness of tumor cells increases with increasing invasion depth, and then the phenomenon of invasion of vascular lymphatic vessels may appear [27, 28]. Studies have shown that tumor invasion depth and lymph node metastasis are independent prognostic factors in determining survival of gastric cancer patients [29], and our study found the same results. Although TNM staging is generally recognized, there are certain limitations. Gastric cancer is highly heterogeneous and prone to recurrence and metastasis, and the TNM staging does not take into account these biological characteristics of gastric cancer, which may cause bias in the assessment of the disease, resulting in different efficacy after treatment [30]. Chmelo et al. believe that the detection of lymphatic, venous and nerve invasion can help improve the precision of the prognosis of gastric cancer patients, and can also help stratify patients with high risk of recurrence and better guide the decision-making process of adjuvant chemotherapy [31, 32]. Although the T and N stages extracted from our data in this study belong to the 8th edition of the AJCC pathological stage of tumors, because they are both stages M1, and the definitions of tumor invasion depth and regional lymph node metastasis have hardly changed after comparing the 7th and 8th editions of TNM stages, the 8th edition TNM stage used in this study will not have a significant impact on the results and limit the use of the constructed prediction model.

At present, the effect of surgery on the prognosis of patients with mGC has been controversial [33, 34]. However, most patients with mGC are generally unable to undergo radical surgery due to non-curative factors, such as distant metastasis, and only undergo non-curative surgeries, such as palliative surgery and dose reduction surgery [35]. Previous studies have suggested that surgery can improve the prognosis of patients with mGC [36–40], and resection of tumor lesions resetion can reduce the tumor burden of patients, prevent or alleviate the occurrence of some complications caused by tumors, and improve the tolerance of patients to chemotherapy to some extent. However, surgical treatment may delay the time of chemotherapy, thereby reducing the effect of chemotherapy and increasing the incidence of adverse events, and surgical treatment may also suppress body immunity, activate and release some inflammatory factors, promote the growth and metastasis of residual tumors, and lead to further disease progression. Furthermore, there are many types of surgeries for patients with mGC due to different diseases, including translational surgery, palliative surgical resection, and the effect of different types of surgery is various, and the prognosis is also different [41]. The prognosis of patients with different surgical outcomes also varies, but the impact of these factors on prognosis cannot be further clarified due to the limited data from the database. Although the results of this study suggest that patients undergoing surgery have a lower risk of cancer-specific death, it does not show that all patients benefit from surgical treatment, and the effect of different surgical types on patient mortality in clinical observation needs to be further studied, so more prospective studies are needed in the future to study the effects of surgery and different surgical types on cancer-specific mortality in patients with mGC.

Although some studies report prognostic factors or models for mGC, few nomograms have been developed for the eighth edition of the AJCC TNM staging system, and the applicability of various biomarkers and inflammatory states in clinical practice remains unclear. On the contrary, the clinical variables included in our nomograms can be measured relatively easily and economically, and as a result, our nomograms are a more economical and practical option.

There are still some limitations to this study. First, this study is a retrospective study of two centers, not a prospective study, and the sample size is relatively small, so there is some selectivity bias. Second, our nomogram only included five clinicopathological factors, and did not take into account the general physical condition, underlying diseases, inflammatory indicators, post-chemotherapy reactions, and surgical complications of the patients, and the specific regimens of radiotherapy were not involved, and the impact of these factors on metastatic gastric cancer needs to be clarified in the future. Third, because the modeling cohort and the external validation cohort use data from different regions of China, geographical differences, ethnic composition and eating habits may have affected the results. Fourth, the median follow-up time differed between cohorts, which could lead to inaccuracies in cancer-specific deaths. Finally, all patients included in the external validation cohort were from a single institution in China with a small sample size, and larger, multicenter prospective randomized controlled trials are needed to validate the validity of nomograms.

## Conclusions

In our study, we identified a series of factors as independent risk factors associated with cancer-specific survival, including differentiation grade, T stage, N stage, without chemotherapy and without surgery. We also identified the impact of chemotherapy regimens and cycles on the prognosis of mGC. Therefore, we developed and validated a nomogram-based prediction suitable for the Chinese mGC population. It helps clinicians to more accurately assess the survival status of patients and further promotes personalized treatment, which has important clinical utility.

## Data Availability

The data presented in this study are available on reasonable request from the corresponding author, Dr. Jun Ren.
